# Pulmonary Hypertension in Pregnancy: A Review

**DOI:** 10.3390/medicina57030259

**Published:** 2021-03-11

**Authors:** Humayun Anjum, Salim Surani

**Affiliations:** 1 Internal Medicine, University of North Texas, Fort Worth, TX 76107, USA; 2 Internal Medicine, Texas A&M University, College Station, TX 77843, USA; Salim.Surani@hcahealthcare.com

**Keywords:** pulmonary hypertension, pulmonary arterial hypertension, pregnancy, right heart catheterization, pulmonary arterial pressure, pulmonary vascular resistance, systemic vascular resistance, cardiac output, right heart failure

## Abstract

Pulmonary hypertension (PH) is a disease, which targets the pulmonary vasculature affecting the heart and the lungs, and is characterized by a vast array of signs and symptoms. These manifestations of PH in pregnancy are highly variable and non-specific hence, it is prudent to have a very keen and high index of suspicion while evaluating these patients. This rare disease can be extremely debilitating and can be associated with a poor overall prognosis. Pregnancy in women with PH puts them at an elevated risk because the physiological changes associated with pregnancy are not well endured leading to even higher morbidity and mortality in these patients. Although there are various modalities for evaluation and workup of PH, right heart catheterization (RHC) remains the gold standard. A mean pulmonary artery pressure (PAP) of more than 20 mm of Hg is considered diagnostic. It is indeed heartening to see that in the past decade many novel therapeutic modalities have emerged and along with a better understanding of the disease process have proved to be promising in terms of reducing the adverse outcomes and preventing death in this population of patients.

## 1. Introduction

Pulmonary arterial hypertension (PAH), which is classified as group 1 PH (Pulmonary hypertension) is defined as an increase in PAP >20 mm of Hg at rest accompanied by an elevated pulmonary vascular resistance (PVR) of >3 Wood units as assessed by RHC as per the sixth World Symposium on Pulmonary Hypertension [1]. In general, women are affected more than men when it comes to PH. Additionally, a vast majority of women affected by PH are young and of childbearing age [2,3]. There is a lot of uncertainty regarding how the physiological changes associated with pregnancy cause a shift in sex hormones. However, it is known that PH in pregnancy leads to a significantly higher mortality rate, between 30–56% [4]. Therefore, expert opinions and guidelines appropriately recommend against pregnancy in PH. However, it is not uncommon to see PH in a pregnant patient, and occasionally it is also seen that patients with PH do get pregnant despite counseling [5].

The purpose of this review is to focus on PAH in pregnancy as most of the data is focused on this subset of patients and there is a significant knowledge gap that exists regarding the effects of sex hormones of pregnancy on the pulmonary vasculature in other WHO groups of PH except for maybe WHO group 3 (hypoxia associated PH) [6]. In this article, we will review the classification of PH, physiological changes that happen in a pregnant patient, the pathophysiology and biology of PH, contraception counseling, and lastly the treatment strategies that are available for these patients. 

## 2. Classification of Pulmonary Hypertension

As we know PH is a general term that is used to describe the high pressure in the pulmonary arteries. Healthy pulmonary arteries are thin-walled and have normal PVR, which is approximately 100–200 dynes/s/cm^−5^. Similarly, the heart in healthy people has a smaller and thinner right ventricle (RV) and has a normal cardiac output (CO), which is approximately 5–6 L/min. As the pressure increases in the pulmonary vasculature, compensatory mechanisms come into play and cause the pulmonary arteries to become constricted and stiff due to abnormal endothelium, which causes a mild increase in the PVR and subsequently a moderate decrease in perfusion. This leads to a hypertrophied right ventricle with normal CO. Further deterioration leads to more cellular proliferation in the wall of the pulmonary arteries leading to obliterative modeling, which in turn leads to an increase in PVR and a decrease in perfusion. At this stage, the RV is dilated, and the CO is severely decreased. Initially, in 1973, PH was classified into two major groups, primary PH if the cause of the disease was known and secondary PH if the cause could not be identified. Now, almost five decades later the classification has expanded and includes five groups, which are outlined in the Table 1.

## 3. Physiological Changes Seen in Pregnancy

**Figure 1:**
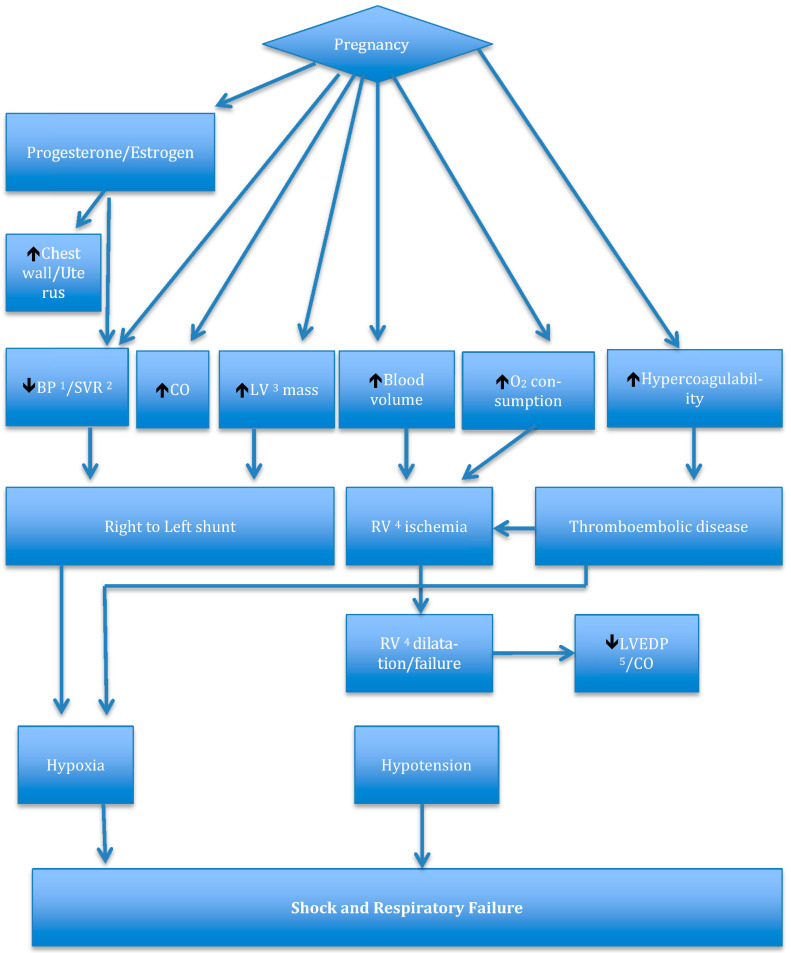
Basic physiological changes seen in pregnancy. ^1^ BP = Blood Pressure, ^2^ SVR = Systemic vascular resistance, ^3^ LV = Left ventricle ^4^ RV = Right ventricle, ^5^ LVEDP = Left ventricular end diastolic pressure.

In a true sense, every organ system is affected by pregnancy. Additionally, the most significant changes are further highlighted in Figure 1. Mechanical changes in pregnancy occur as a result of the increase in the size of the uterus and the chest wall, which are hormone mediated. This can cause the Functional residual capacity (FRC) to decrease by at least 20% [7]. Additionally, the enlargement of the uterus can affect the perfusion in the inferior vena cava particularly in the supine position. In addition to these mechanical changes, the sex hormones increase significantly during pregnancy [8,9,10,11]. The very first hormone that peaks around the 10^th^ week of gestation is human choriogonadotrophin (hCG) [12]. Subsequently, progesterone and estrogen are the other two major hormones that remain elevated for the rest of the gestational period and fall sharply immediately following delivery [13]. These changes due to a shift in the hormonal balance can have a dramatic effect on the cardiopulmonary system, which is further reviewed below.

Cardiovascular system and pregnancy: The most noticeable change is the increase in plasma volume. Generally, this starts early in the pregnancy and peaks just before delivery to about 50–70% of the pre-pregnancy levels. This is mainly hormone-mediated and happens due to the vasodilatory effects of the sex hormones leading to under filling of the atrium, activation of the renin-angiotensin system, decrease in the levels of the natriuretic peptide, and subsequently retention of sodium and water. Additionally, this amounts to about an additional 6–8 L of fluid [14,15,16,17,18]. The erythropoietin production is increased in pregnancy but only by 20–30% so the rise in red cell mass is much less than the increase in plasma volume and it leads to dilutional anemia which is important for the facilitation of the perfusion of the fetoplacental unit [19,20]. Increased risk of pulmonary emboli and pulmonary arterial thrombosis happens due to the hypercoagulable state. The mechanical forces of pregnancy cause the cardiac axis to shift leftwards and also causes stretching of the chambers, which can lead to benign arrhythmias and an increase in the heart rate by at least 15–20 beats/minute [21,22,23,24,25]. An increase in cardiac output (CO) is typically seen in the first trimester. Interestingly the left ventricular ejection fraction (LVEF) is unchanged in pregnancy [26]. Progesterone and estrogen lead to vasodilation. This is thought to be due to increased nitric oxide (NO) and prostacyclin production. This results in a 40% decrease in systemic vascular resistance (SVR). It is prudent to realize that although the blood pressure decreases somewhat in the first 2 trimesters, it increases to the pre-pregnancy baseline levels in the third trimester [27]. 

Pulmonary system and pregnancy: A normal pulmonary system can easily accommodate the demands of pregnancy just like the systemic circulation. Similar changes happen in pregnancy as well. It is well known that oxygen consumption increases at least by up to 20% in pregnancy [28]. This leads to an increase in tidal volume (TV), which is primarily mediated by progesterone. Although, it is important to note that the respiratory rate (RR) remains the same. Dyspnea due to this increase in alveolar ventilation is seen in a vast majority of these patients [29]. The result is a change in the acid-base status causing respiratory alkalosis and a compensatory metabolic acidosis. The normal values of pH, PaCO_2_, and PaO_2_ are 7.39–7.45, 25–33 mm Hg, and 92–107 mm Hg respectively [30,31]. Another challenging situation that is seen in pregnant patients is airway edema [32]. A lot of studies have pointed towards this finding and it is also noted that this phenomenon is not dependent on the duration of labor and/or the number of fluids given to a pregnant patient [32,33]. This in combination with the reduced FRC in a setting of PH can make endotracheal intubation very tricky and should be planned accordingly. As it is seen in the systemic circulation the pulmonary vascular resistance (PVR) decreases and due to the opposing effects of the increased CO and decreased PVR the PAP remains unchanged [1]. All these normal regulatory mechanisms are impaired in a setting of vascular pathology. This causes increased afterload for the right ventricle (RV) and eventually leads to RV failure and this may be more so in the postpartum period as the CO increases even more. 

## 4. Changes Seen in the Cardiovascular System during Labor

The cardiovascular system in particular is prone to changes during labor and the peripartum period, as these changes happen rapidly in contrast to pregnancy and are due to the change in volume status and shifts in the intrathoracic pressure. As mentioned previously, the increase in CO is much more pronounced during this period and this along with the increase in SVR can put a patient with a compromised vascular bed such as PH in a very tenuous situation [34,35]. 

## 5. Pathophysiology of Pulmonary Hypertension

The two most important processes that lead to PH are increased pulmonary vascular resistance and venous pressure. The increase in vascular resistance occurs because of a proliferative process due to alterations in the vaso-affective molecules. Thromboxane and endothelin-1 are vasoconstrictors and prostacyclin and nitric oxide are vasodilators. The enhanced activity of the vasoconstrictors and reduced activity of the vasodilators cause this imbalance. Increasing vasoconstriction leads to proliferation, fibrosis, and microthrombi in the pulmonary arterial system [36]. The increase in pulmonary venous pressure is usually a result of disorders affecting the left side of the heart. It is well known that patients with idiopathic pulmonary arterial hypertension (IPAH) having an underlying genetic predisposition and patients with hereditary pulmonary arterial hypertension (HPAH) have an inheritable genetic mutation of which BMPR2 is the most common one, seen in about 80% of the cases of HPAH. It is usually inherited in an autosomal dominant fashion [37]. Caveolin 1 (CAV1), KCNK3, and EIF2AK4 are the other mutations that are responsible for the rest of the 20% of cases of HPAH [38,39,40]. Regardless of the cause, the final pathway in PH is RV strain or failure. In acute settings, RV dilatation is seen whereas in chronic PH RV hypertrophy is noted. The hemodynamic consequences of this impairment are tremendous as it may cause increased oxygen demand, reduced CO due to impairment of LVEF, and hypoperfusion of the right coronary artery leading to RV ischemia [41,42,43]. 

## 6. Biological Principles of Pulmonary Hypertension in Pregnancy

After reviewing the basic physiological changes associated with pregnancy and pathophysiology of PH it should not be difficult to determine that the most pressing challenge in patients with PH during pregnancy is the reduced ability to accommodate the significant changes that occur in the cardiovascular and pulmonary system due to a compromised vascular bed. 

As mentioned previously, the pulmonary vasculature in pregnancy responds to these high demands by dilating and creating more collateral vessels, which happens due to perfusion of non-perfused vessels. This leads to a decrease in PVR. One study has suggested that the vasculature in the pulmonary vessels in women is more distensible than in men. However, its significance to pregnancy is not very clear [44]. It is worth pointing out that even though the direct role of sex hormones in the pulmonary vasculature is unclear there is enough evidence to show that these sex hormones do affect them [6,45,46,47,48,49]. Inherently, several hormones are pathologically elevated in patients with PAH which are beyond the scope of this discussion and the bottom line is that pregnancy has the potential to enhance their effects. Additionally, all of these effects are further exacerbated during weeks 20–24, the early part of the third trimester, and the postpartum period as these times reflect the stages where most significant hemodynamic changes happen [50]. The inherent effect of the sex hormones and their metabolites, and in particular of estrogen as mentioned above is still unclear but it is postulated that there is a lack of substrate for the sex hormones to initiate their vasodilator properties and that estrogen can worsen the pulmonary vascular bed remodeling process [51,52,53,54]. So, is it due to the latter that there is worsening of PH in pregnancy, or is it due to the above-mentioned changes in the cardiovascular and respiratory physiology? This remains unclear and undetermined and further studies will be needed. Primary pulmonary hypertension of the newborn (PPHN) is an interesting subset of group 1 PH. The risk factors for PPHN are maternal age, smoking history, parity status, BMI, and maternal use of selective serotonin release inhibitors (SSRI) [55,56]. However, the association between SSRI and PPHN remains controversial [57].

## 7. Role of Counseling Regarding Contraception in Patients with Pulmonary Hypertension

This may be the most difficult discussion that a physician needs to have with a patient. As soon as the patient is diagnosed with PH, she should be counseled regarding avoiding pregnancy. This cannot be emphasized enough as multiple studies in the literature have shown that these women are not informed about the importance of contraception and even those who are, are not given a detailed description of the available options [58,59]. This becomes increasingly important when a genetic condition is suspected. It is known that genetic mutations in PAH have been identified in IPAH and familial pulmonary arterial hypertension (FPAH), anorexigen-associated PAH, pulmonary veno-occlusive disease, and PAH associated with congenital heart disease. This practice is crucial as it may have a huge impact on other family members and future generations. In North America and Europe clinical testing for these genetic mutations is available and must be utilized judiciously. However, prior to proceeding with genetic testing education and counseling should be made available to ensure all the clinical and psychosocial aspects which usually accompany all genetic diseases are addressed as they impact the patient and their families in a distressing way. For obvious reasons estrogen-containing contraceptives should not be recommended as they will increase the risk of venous thromboembolism (VTE) and are detrimental for the pulmonary vasculature. Furthermore, estrogen can contribute to the pathogenesis of PAH in pregnant patients. Progestin-only contraceptives seem to be the safest choice in patients who are not good candidates for the permanent sterilization due to various underlying reasons, with a caveat that some literature supports the fact that injectable formulations may be associated with an increased risk of VTE [60]. Intrauterine device (IUD) could be the other choice. Traditionally, IUDs can be copper IUD and progestin-releasing IUD. However, it is of utmost importance to remember that manipulation of the cervix during the placement of these IUDs can trigger a vasovagal response, which can be detrimental in these patients with PAH. Therefore, extreme caution is usually advised. Additionally, IUDs can be associated with an increased risk of pelvic infections so that needs to be taken into account as well. Overall, progestin-releasing IUD is favored over copper IUD due to a decreased rate of menstrual blood loss. Barrier methods are not routinely recommended due to high failure rates. Lastly, permanent methods such as tubal ligation or a device implanted into the fallopian tube can be considered as well on a case-to-case basis and institutional recommendations as they seem to be the perfect solution given the risks and uncertainties associated with other methods of contraception [61]. Emergency contraception can be achieved utilizing the above-mentioned hormonal methods versus placement of copper IUD. Additionally, be aware that the patients who are taking bosentan will need a higher dose of emergency contraception due to drug interactions. 

## 8. The Treatment of Pulmonary Hypertension in Pregnancy

In this section, we will review four different classes of medications, the role of anticoagulation, and the overall management of pregnancy in patients with PAH.

### 8.1. PAH Directed Medications in Pregnancy

There are four major classes of medication for the treatment of PAH, prostaglandins, phosphodiesterase 5 inhibitors, endothelin receptor antagonists, and soluble guanylate cyclase stimulators. Also, calcium channel blockers may have a role. Let us review them in more detail. Additionally, Table 2 below lists the pregnancy category of these medications. Pregnancy category B is based on animal reproduction studies that have failed to demonstrate a risk to the fetus. But, there are no adequate and well- controlled studies done in pregnant women. Pregnancy category C is based on animal reproduction studies that have shown an adverse effect on the fetus. But, once again there are no adequate and well-controlled studies done in pregnant women. Lastly, pregnancy category X is based on studies in animals or humans and have demonstrated fetal abnormalities and/or there is positive evidence of human fetal risk based on adverse reaction data from investigational or marketing experience and the risks involved in use of the drug in pregnant women clearly outweigh potential benefits.

1. Phosphodiesterase 5 inhibitors (PDE5): If a patient becomes pregnant PDE5 can be continued. In general, tadalafil is less favored due to lack of data. Most of the literature supports the use of sildenafil with prostaglandins [62,63]. Additionally, generally speaking, monotherapy with PDE5 inhibitors is only for patients who have normal RV function and are in WHO functional class (FC) I or II or who refuse prostaglandin therapy [62,64]. Patients who are on monotherapy need a very close follow-up. 

2. Prostaglandins: If a patient becomes pregnant prostaglandins can be continued. However, if the patient is on this class of medications before the pregnancy it is an indicator of severe disease. The usual indications for starting prostaglandins are WHO FC III or IV and impaired RV function because they are not only potent vasodilators but at the same time enhance the RV function [65,66]. Pregnant patients with WHO FC IV and with severely reduced RV function are candidates for parenteral prostaglandins. Additionally, parenteral formulations can also be considered in pregnant patients who are not improving or worsening with standard therapy. The preferred choice is IV epoprostenol. Despite all of this, be aware that mortality remains high. Some of the medications in this class are also available in an inhaled formulation and, in that case, inhaled iloprost is preferred [65,66,67].

3. Endothelin receptor antagonists and soluble guanylate cyclase stimulator: All the medications under these classes are category X and should be discontinued as soon as the patient becomes pregnant. Note that Riociguat is now indicated for the treatment of chronic thromboembolic pulmonary hypertension (CTEPH).

4. Calcium channel blockers: They are generally considered safe in pregnancy and have a role in patients who are considered vasoreactivity based on the response to inhaled vasodilators such as nitric oxide. Additionally, one study did show that these patients who are vasoreactive respond better than their counterparts [68]. However, be aware that they should not be considered if the patient is WHO FC IV or has RV dysfunction [68].

### 8.2. Anticoagulation in a Pregnant Patient with PH

Anticoagulation has a role in many forms of PH, particularly IPAH, HPAH, some forms of congenital heart disease associated PH, and most importantly in CTEPH [69]. Generally, for pregnant patients who need to be on chronic anticoagulation low-molecular-weight-heparin (LMWH) is usually used. However, consider changing to unfractionated heparin close to the delivery date, as it is easier to discontinue if needed. Warfarin is a teratogen and is contraindicated. Data regarding the new oral anticoagulants is limited and they are all pregnancy class C category, so they are not recommended [69,70,71].

### 8.3. General Principles of Management of PH in Pregnant Patients

As it has been established, pregnancy in patients with PH can lead to adverse outcomes both for the fetus as well as the mother so it is recommended to offer termination of pregnancy. This is a shared and informed decision-making process and is influenced by a multitude of factors. The best time to terminate the pregnancy is in the first trimester and it can be accomplished via surgical evacuation or use of medications if surgery is not favored [70]. It is important to understand that patients with PH are in general at a higher risk as compared to the general population when it comes to termination of pregnancy and hence it should be performed in experienced centers [72]. The safest surgical procedure is uterine dilatation and evacuation, and the medical abortion is done using prostaglandin E1, E2, or misoprostol [73]. 

Some patients with PH will choose to continue with pregnancy despite the known risks and complications. Given the complicated nature of PH and in particular, in a pregnant patient multidisciplinary team approach is favored. Follow-ups during the first, second, and third trimesters should be monthly, two weekly, and weekly respectively. The patients are advised to lie in a lateral position to avoid caval vein obstruction and other general principles of right heart failure remain the same for pregnant patients and include fluid and salt restriction, diuretics, and supplemental oxygen if needed [74]. Furosemide is the preferred diuretic; spironolactone is pregnancy category D and should be avoided [70]. 

Two notable diseases that can lead to acute pulmonary hypertension in pregnant patients are pulmonary embolism (PE) and amniotic fluid embolism (AFE). Any thromboembolic disease in a pregnant patient is usually treated with either unfractionated heparin or LMWH. The latter can sometimes be tricky as it may require monitoring of anti-factor Xa levels. As mentioned previously Coumadin is teratogenic and contraindicated [75]. Now, anticoagulation by itself may not be sufficient if it is a case of massive pulmonary embolism. The options “in that case” are thrombolytics, embolectomy and IVC filter placement. Pregnancy is listed as relative contraindication when it comes thrombolytics however; it has been used in this patient population. However, the evidence is restricted only to case reports [76,77]. Hence, the decision to administer thrombolytics to a pregnant patient is a crucial one and will need to be assessed on an individual basis. Embolectomy on the other hand was shown to be associated with a higher rate of fetal loss as compared to thrombolytic therapy and should be used only in case of a catastrophic situation [78]. The indications for IVC filter placement in a pregnant patient are the same as in non-pregnant patients [75]. Generally speaking, the course of AFE is biphasic. The initial insult leads to vasospasm and induction of PAH which leads to right ventricular failure and subsequently cardiac arrest. AFE is associated with a very high mortality rate and a vast majority of patients do not survive this event. However, the ones that do usually have resolution of PAH and develop left ventricular failure. The treatment is usually supportive and focused on preventing further hypotension and hypoxia [79]. Recently, extracorporeal membrane oxygenation (ECMO), intra-aortic balloon pump (IABP), and ventricular assist device (RVAD) have been mentioned in some studies as part of the treatment of AFE as the goal is to prevent respiratory and circulatory collapse in these patients [80,81]. If hypotension and circulatory collapse seem to be the predominant issues and oxygenation is stable while being on mechanical ventilatory support then IABP and RVAD would be the go to options. IABP as we know increases coronary perfusion and leads to improvement in cardiac contractility. RVAD helps unload the RV because in acute PAH as the ratio of PAP as compared to systemic arterial pressure (SAP) keeps increasing it this leads to decreasing CO. Additionally, theoretically it would make sense that improvement in the flow and perfusion due to improved hemodynamics would result in improvement of oxygenation as well. On the other hand, if both oxygenation and hemodynamics are significantly impaired and not improving with conventional therapy then ECMO should be strongly considered as it can not only help with unloading of RV but at the same time can help with oxygenation. Additionally, it is recommended to firmly think about the fetal delivery options if it has not already happened to prevent further complications and to facilitate supportive treatment as mentioned above.

It is advisable to opt for an elective cesarean section as soon as it is determined that the fetus is viable which is usually between 34–36 weeks because vaginal delivery can be complicated in such patients due to hemodynamic consequences and can be associated with significant morbidity and mortality [82].

The postpartum phase and particularly, the first week is very crucial as it is well known that most of the pregnant patients with PH acutely decompensate during this time due to the hemodynamic changes. Decompensated heart failure, sudden cardiac death, and pulmonary thromboembolism seem to be the most common causes of death in such patients [70,83]. 

Additionally, lastly, it is important to note that vagal responses in patients with PH can be extremely consequential as they cause a significant drop in CO. This, is more concerning in pregnant patients as their RV is very preload dependent and the compensatory responses are not that strong. Multiple normal and physiological human responses can trigger vasovagal syncope and the clinicians need to have heightened awareness about these triggers.

**Figure 2:**
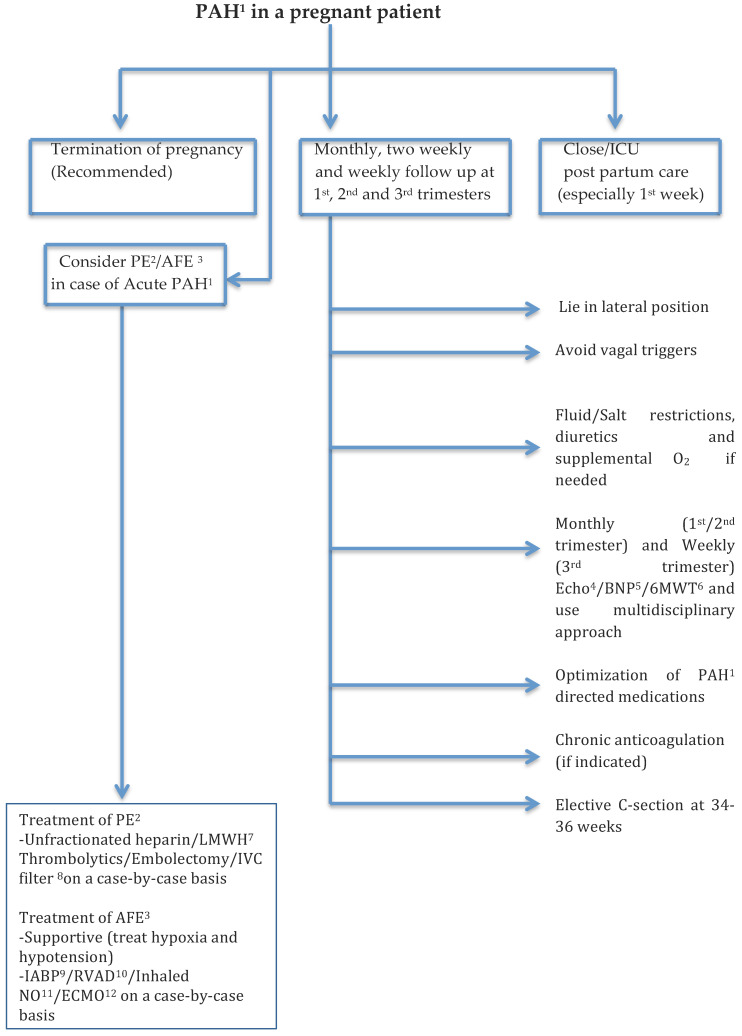
General scheme for management of PAH in a pregnant patient. ^1^ PAH = Pulmonary arterial hypertension, ^2^ PE = Pulmonary embolism, ^3^ AFE = Amniotic fluid embolism, ^4^ Echo = Echocardiogram, ^5^ BNP=Brain natriuretic peptide, ^6^ 6MWT = Six minute walk test, ^7^ Low molecular weight heparin, ^8^ Inferior vena caval filter, ^9^ Intra-aortic balloon pump, ^10^ Right ventricular assist device, ^11^ Inhaled nitric oxide, ^12^ Extracorporeal membrane oxygenation.

Figure 2 summarizes the management of PAH in pregnant patients.

## 9. Conclusions

PH and in particular PAH is associated with increased morbidity and mortality in the general population and this is amplified in a pregnant patient. It is important to understand the basic principles of PH and physiological changes associated with pregnancy and have in-depth knowledge of all the available modalities used in the treatment of this devastating disease as managing these complex patients can be extremely challenging. Last and not least, it is crucial to have a multidisciplinary team-based approach involving maternal-fetal medicine obstetricians, neonatologists, PH specialists, cardiologists, and anesthesiologists while ailing these patients to good health.

## Figures and Tables

**Table 1:** Classification of Pulmonary Hypertension.

Group	Classification	Causes/Etiologies
Type 1	Pulmonary Arterial Hypertension (PAH)	1. Idiopathic PAH
		2. Hereditary/Familial PAH
		3. Associated PAH (Connective tissue diseases, Congenital heart disease, Portal hypertension, HIV, Drugs and Toxins, Pulmonary veno occlusive disease)
		4. Pulmonary hypertension of the newborn
Type 2	PH due to cardiac disease	1. Left sided systolic or diastolic heart failure
		2. Valvular heart diseases
Type 3	PH due to lung disease and/or hypoxia	1. Chronic obstructive lung disease
		2. Interstitial lung disease
		3. Sleep disordered breathing
		4. Developemental abnormalities
Type 4	Chronic thromboembolic PH	Thromboembolic and non thrombotic obstruction of pulmonary arteries
Type 5	PH due to miscellaneous causes	1. Sarcoidosis
		2. Histiocytosis X
		3. Chronic hemolytic anemia

**Table 2:** Pregnancy category for PAH medications.

Classification	Medication	Pregnancy Category
Prostaglandins	Epoprostenol	B
	Treprostinil	B
	Iloprost	C
Phosphodiesterase 5 inhibitors	Sildenafil	B
	Tadalafil	B
Endothelin receptor antagonists	Ambrisentan	X
	Bosentan	X
	Macitentan	X
Soluble guanylate cyclase stimulator	Riociguat	X
